# Health Tract No. 345

**Published:** 1868-11

**Authors:** 


HEALTH TRACT NO. 345.

### SAVING LIFE.

Once upon a time a Tanner proved, very satisfactorily to himself, that leather was the most valuable thing in the world. Will not damp feet give coughs, colds and consumptions by the million, and don't leather keep the feet from getting damp? It may be said with greater truth that doctors, physically speaking, are the most useful class of men on the face of the earth; albeit, they are made a jest and butt of by everybody—until sickness comes, and then the physician's coming is greeted with a deeper satisfaction than that of Royalty.

By finding out what are the causes of disease, which is the first step for a cure—and the want of bread and butter impelled him to that—he has forestalled disease in millions of cases; which he was not obliged to do, but his sense of humanity prompted him to do this · and this has been going on for centuries, and see the result.

The average duration of life in Switzerland, three hundred years ago was only twenty-one years, it is now forty-five years, more than double, arising from the greater intelligence of the people as to habits of eating, drinking and sleeping, and the modes of avoiding disease; France and England show an equal increase in the duration of life. But medical men are not satisfied with these results, and have been pursuing their investigations most industriously in the direction of local and personal cleanliness and the ventilation of our dwellings and bed chambers, in which we spend one entire third of our existence, and it is during the impassive condition of sleep that we are most liable to the influence of the causes of disease, and medical men have shown that disease and premature death are avoidable in an incalculable number of instances. It is demonstrated, that in Philadelphia seven thousand persons die annually by unnecessarily breathing an impure air, and as each death involves an average of thirty days' sickness, or five hundred and sixty years of labor of one person besides.

The health of a town can be measured by its cleanliness; on the approach of Cholera in Worcester, England, in 1849, the authorities determined to test the value of cleanliness in resisting the disease, as the physicians insisted; every lane, alley and cellar was swept, washed and cleaned most scrupulously, and "not a house was entered by the disease and the town was saved in the midst of the most frightful devastation."

In 1857, one out of twenty-five died in New York City; in the same year in London, there was one death in forty-two; that is, nearly double the number of deaths took place in New York as compared with London, and there is no reason why New York should not be as healthy as London; if it is kept as clean, it will be, if the authorities will carry out the measures of cleanliness and ventilation which Griscom, Harris and other medical men, whose scientific investigations have so pre-eminently qualified them to speak with authority on these subjects, have advised. Forty per cent of those who die in Philadelphia and nearly one-half in New York perish unnecessarily early from breathing bad air.

